# Causal Factors Contributing to Youth Cyberbullying in the Deep South of Thailand

**DOI:** 10.3390/children11070790

**Published:** 2024-06-28

**Authors:** Kasetchai Laeheem

**Affiliations:** Faculty of Liberal Arts, Prince of Songkla University, Hat Yai District, Songkhla 90110, Thailand; kasetchai.la@psu.ac.th

**Keywords:** cyberbullying, negative upbringing, the influence of personal violence, the influence of media violence, negative mental traits

## Abstract

Background: Violence against each other via social media has increased and caused cyberbullying that can happen anytime through electronic communication tools that everyone can access easily. Cyberbullying is sending, posting, and sharing negative, harmful, and false information about another, causing embarrassment online on social media. Objectives: This study aims to investigate causal factors contributing to youth cyberbullying in Thailand’s deep south. Methods: A cross-sectional survey was conducted among 340 youths in Thailand’s deep south, consisting of 220 males and 120 females. The subjects were divided by age range: 22–23 years old (40.6%), 24–25 years old (26.8%), 18–19 years old (17.1%), and 18–19 years old (15.5%). The data were analyzed with structural equation modeling (SEM). Results: The results were that the model of the causal factors resulting in youth cyberbullying in the deep south of Thailand was consistent with the empirical data (the relative chi-square (χ^2^/df) was 1.77). The goodness-of-fit index (GFI) was 0.95. The root-mean-square error of approximate (RMSEA) was 0.049). Cyberbullying was positively influenced directly and indirectly by negative upbringing, the influence of personal violence, and the influence of media violence at a statistically significant level of 0.001, with total effect sizes of 1.13, 0.74, and 0.64, respectively. Additionally, cyberbullying was positively influenced directly by negative mental traits with a statistically significant level of 0.05 and a total effect size of 0.17. Conclusions: This study suggests that the results could be beneficial in concretely forming policies and strategies to prevent and mitigate the problem of youth cyberbullying.

## 1. Introduction

Cyberbullying affects the way of life of people of all groups, particularly youth in Thailand, who rank highest in internet usage compared to other groups [1]. Youth neither screen nor carefully select modern technology for use in daily living and this has led to new forms of youth violence, especially among those who are frequent users of social networks, chats, and e-messages [2]. At the same time, violence against each other via social media has increased and caused cyberbullying that can happen anytime through electronic communication tools such as computers and cell phones that everyone can access easily [3]. Thus, Internet addiction plays a mediating role in the association between cyber victimization and cyberbullying [4]. Cyberbullying is a modern form of aggression, occurring among youth, involving the use of swift and easily accessible information technology that takes place over digital devices like mobile phones, desktop computers, laptop computers, and tablet computers. Cyberbullying is sending, posting, or sharing private, negative, harmful, and false information about another, causing embarrassment through SMS, text, pictures, and apps, or online in social media including Facebook, Line, Instagram, YouTube, and Twitter, or through gaming where people can view, participate in, or share content [3,5]. Cyberbullying can be classified into five categories: (1) Verbal aggression: This includes harmful verbal actions in the cyber world, such as offensive language, derogatory remarks, mocking embarrassing behavior, ridiculing physical appearance, and dishonorable challenges. (2) Defamation: This involves spreading shame, creating hatred, disseminating manipulated images, sharing embarrassing photos, and releasing unfavorable news. (3) Impersonation: This involves using another person’s name in conversations to harm them, create embarrassment, manipulate their images, seek personal gain using their name, or borrow money or possessions in their name. (4) Privacy breach: This involves revealing or forwarding parents’ names, revealing or sharing inferiority complex, embarrassing secrets, sharing personal information without consent, and exchanging secrets with third persons. (5) Blocking or deleting: This involves removing disliked individuals from one’s friend list, blocking people disliked within a group, forcing friends to delete disliked individuals, and compelling friends to block those disliked within a group. These actions occur rapidly and effectively through online social networks, providing a platform for everyone to freely perceive and express opinions. Meanwhile, the victims, unable to respond, experience stress, pain, embarrassment, and a loss of confidence in their social existence [3,5].

A comprehensive review of the related literature revealed that the impact of cyberbullying has intensified, emerging as a silent threat to Thai youth and contributing to a range of social issues [6,7]. Research findings indicate that cyberbullying significantly correlates with suicide, drug use behaviors, and unsafe sexual practices among young individuals in addition to mental distress leading to daily life concerns [8,9]. Cyberbullying manifests both direct and indirect effects on the targeted person. Direct effects include gossip, vulgar language, defamation, threats, and verbal harassment resulting in emotions such as anger, frustration, stress, anxiety, and embarrassment. Indirect consequences impact mental health, physical wellbeing, depression, social relationship difficulties, and even suicidal ideation [9,10].

Recent studies on the prevalence of cyberbullying among youth found that cyberbullying occurs at a significantly high rate. For instance, it accounts for 51% of the central part of Thailand [11], 27% in Korea [12], 40% of the United States [13], 23% among youth in the Midwestern states of the United States [9], 54% of a university in the United States [14], 40% in England [15], 53% in Germany [16], 29% in Israel [17], and 39% in Belgium [18]. One crucial causal factor contributing to cyberbullying stems from negative family upbringing. Whether it is overly strict and controlling parenting, neglectful parenting with minimal supervision, or indulging parenting that excessively caters to the child’s desires and fosters self-centeredness, all forms of negative family upbringing can lead to feelings of sadness, repression, and emotional distress among youth. These negative emotions often result in inappropriate behaviors, including cyberbullying [19,20,21]. Previous studies on its prevalence for cyberbullying and cyber victimization showed that more than a third of youth across 30 countries reported rates ranging from 6% to 46% for cyberbullying and from 14% to 58% for cyber victimization [22]. This shows increased trends in prevalence rates of cyberbullying and cyber victimization in many Asian countries and Australia and decreased trends in Western countries [23].

Additionally, the causal factors contributing to cyberbullying include both influences of aggressive behavior from individuals and the media. These include influences from parental aggression, peer aggression, internet idol aggression, film violence, video game violence, and news violence. These factors are linked to the absorption and imitation of negative behaviors from individuals and the media. Specifically, youth tend to imitate those who are close to them and with whom they have frequent interactions. Consequently, these behaviors manifest in their interactions with people around them in their daily lives. Studies found that aggressive individuals and media content depicting threats and violence are often associated with youth’s violent behaviors and cyberbullying [24,25].

Moreover, there are negative mental factors that contribute to cyberbullying. These include feelings of frustration, anxiety, and paranoia. Such emotions often trigger anger, fear, pressure, irritability, and quick mood swings. Consequently, individuals may exhibit inappropriate verbal expressions and actions. Research has found that the process of absorbing and accumulating past experiences plays a role in developing negative mental traits related to unmet emotional needs and the inability to act or express oneself as desired. These traits may arise due to obstacles, resulting in feelings such as frustration, resentment, irritability, anxiety, worry, distress, and apprehension. Subsequently, these symptoms transform into emotional frustration, anxiety, and paranoia, which tend to correlate with aggressive behavior and cyberbullying as an outlet for the emotions experienced at that moment [26,27,28,29].

The context of this study is the deep south of Thailand; Pattani, Yala, and Narathiwat, which have a combined population of between 1.8 and two million people, of whom more than 1.5 million are ethnic Malays who profess the Islamic faith. This region has suffered chaos and an ongoing crisis as a result of violence that has lasted for over 20 years. The issue of cyberbullying in this area is, therefore, complicated as it is influenced by other problems affecting every aspect of the lifestyles of youth residing in the area [30,31]. The violent situation in this area negatively impacts youths’ feelings, causing them to feel anxiety and fear about ongoing events. This is exacerbated by a negative upbringing, the influence of personal violence, the influence of media violence, and negative mental traits [20,32]. The process can be considered a form of individual learning that results from exposure to harsh environments with negative influences that cause young people to imitate behavior based on accumulated experience. Moreover, such experiences of violence also influence the attitudes of young people, who tend to think that humans can solve some problems better through violence than other methods [19,20].

It is evident that cyberbullying poses a silent threat that deserves attention and awareness regarding its ensuing impact. Consequently, urgently studying causal factors through a thorough analysis is essential for preventing cyberbullying among youth. Therefore, the objective of this research is to explore the causal factors influencing cyberbullying in young individuals. The findings from this study will benefit both individuals and directly related organizations in effectively managing the issue of cyberbullying before it escalates into a societal problem. Collaboratively, we can seek solutions to timely address this pressing concern.

## 2. Literature Review and Conceptual Framework

### 2.1. Cyberbullying

Cyberbullying is one dimension of violence that can indicate behavior affecting individuals’ daily lives in the current era. Cyberbullying refers to the use of technology via the internet by individuals to harass others to cause negative feelings in the victims. It can be classified into five categories: (1) Verbal aggression or insults: This involves messages that take the form of hostility, aggression, intimidation, insults, sarcasm, and insults about skin color or other physical characteristics that demean others, including messages of gossip and repeated use of vulgar and derogatory words against others to express dissatisfaction. All these constitute cyberbullying. (2) Defamation of others online: This aspect involves individuals posting harmful content, including false information or information that damages another person’s reputation. This can occur in the form of text messages and actions through websites or popular programs. Victims of cyberbullying may find it challenging to delete such information, especially if they have never accessed that false information. Additionally, cyberbullying includes manipulating images and videos without permission and posting, sharing, or forwarding them online. (3) Impersonation of others online: This involves falsely claiming to be someone else by posting personal information or various data. Due to the nature of the internet, it is relatively easy to imitate others, and it is difficult to determine whether someone has posted something that lacks credibility. This can also include pretending to be someone else through chat rooms and other programs. (4) Disclosure of others’ private information or forwarding it online: This involves taking others’ private information and sending it to other programs that can be posted on social networks without the permission of the persons concerned. This includes information that may cause damage and embarrassment that is considered a deliberate deception that may or is likely to harm others. (5) Deleting or blocking others from online groups: This involves deleting and blocking others from online chat groups through various popular programs, websites, or gaming applications via mobile phones, computers, or other devices. Research on cyberbullying victims or those excluded from cyber groups indicates that deleting or blocking someone within an online group is one of the most effective forms of cyberbullying [3,21,33,34,35].

### 2.2. Negative Upbringing and Cyberbullying

Negative upbringing is a process of indoctrination that results in poor behavior in children who lack discipline, do not know how to control themselves, and are without responsibility [21,32]. Thus, the family’s upbringing style plays an important role in the children’s personality, temperament, behavior, and development. Inappropriate upbringing can affect children’s personalities and their problem-solving skills [20,28]. A review of the literature revealed that negative parenting in families, including overly controlling, permissive, and excessively indulgent parenting styles, often leads to stress, anxiety, and frustration in adolescents. Consequently, individuals feel the need, and attempt, to find ways to express and release these emotions. The excessively negative format of parenting tends to result in most cases of deviant behavior and cyberbullying among young people [19]. The relationship between upbringing and cyberbullying among adolescents has both direct and indirect implications [3,33]. Negative parenting practices significantly impact adolescents, leading to feelings of sadness, repression, and a diminished sense of self-worth in both themselves and others. These effects, in turn, influence cyberbullying behavior [36]. Moreover, negative parenting prevents adolescents from social adjustment, results in academic failure, diminishes life goals, erodes self-esteem, strains peer relationships, and hinders effective stress management and problem-solving. Consequently, undesirable behavioral manifestations emerge [19,37]. Therefore, overly controlling, permissive, and excessively indulgent family upbringing styles have both direct and indirect negative effects on cyberbullying behavior among young people.

### 2.3. The Influence of Personal Violence and Cyberbullying

The behavior of close and favorite people is one factor that can affect the behavior of young people. Prolonged exposure to violent events involving their parents, friends, and loved ones, whether directly or indirectly, affects youth’s behavior, social adaptation, and attitude in life. This often results in young people perceiving violence as normal, leading them to imitate such aggressive behavior in their daily lives. Consequently, this imitation becomes a significant factor contributing to various forms of bullying. The violent environment where parents, friends, and their favorite people use violence also has a psychological effect on young people. Feelings of confusion, anxiety, insanity, and frustration emerge, leading to various forms of deviant behaviors. As a result, youth often vent their emotions through physical expressions and actions in the cyber world without much consideration for affecting themselves and others [21,32,38]. The influence of parents, friends, and their favorite individuals has a causal relationship with cyberbullying and offenses, drug use, and other deviant behaviors [24,39]. Aggressive behaviors and conflicts between parents, friends, and their favorite people are behaviors directly experienced by youth that affect their emotional and mental state. These are factors contributing to emotional problems and eventually lead youth to behave violently and bully others in their daily lives. Additionally, it reinforces unconscious aggressive behaviors among young people [24,40]. Therefore, the aggressive influence of individuals—ranging from parents and friends to those admired by youth—directly impacts negative psychological traits. Additionally, it has both direct and indirect effects on youth cyberbullying.

### 2.4. The Influence of Media Violence and Cyberbullying

The aggressive impact of media serves as one significant causal factor influencing individuals’ behavior, particularly among young people. Human behavior, shaped by social learning theory, often results from direct experiences and observations of role models. Children learn aggressive behaviors and cyberbullying tendencies by observing violent content in various media forms, including movies, games, and news [20,21,41]. Media violence affects emotions, beliefs, attitudes, and perceptions of normalcy among youth. Consequently, young individuals may imitate violent behavior, assuming it is commonplace. The influence of media violence—whether through visual or auditory content—has a relationship with youth’s behaviors [42,43]. Forms of violence portrayed in media—such as movies, games, and news—directly affect young people’s mental wellbeing. Exposure to violent content, whether through celebrity models or explicit depictions, leads to confusion, anxiety, and frustration among youth. Consequently, they may imitate these aggressive behaviors, resulting in various forms of cyberbullying [20,21,25]. Thus, media violence has a direct influence on negative mental traits and significantly impacts youth cyberbullying behavior, both directly and indirectly.

### 2.5. Negative Mental Traits and Cyberbullying

Negative mental traits originate from the mind and are often shaped by a person’s environment. This factor plays a crucial role in generating emotions that remain unaddressed. Individuals encounter situations that impact their mental wellbeing, leading to feelings of discomfort, resentment, grievance, anxiety, and even insanity—essentially, wounds within the mind. These emotional wounds tend to result in various forms of violent behavior [20,21,44]. Negative mental traits, such as frustration, anxiety, and insanity, often stem from unmet desires and wants. These unfulfilled wishes emotionally trigger negative feelings within the mind, including anger, negative language, actions, and gestures. Over time, these accumulated experiences shape an individual’s behavior. Consequently, they play a crucial role in the expression of inappropriate conduct [27,34]. In addition to the foundational aspect of cyberbullying, which partly stems from frustration, anxiety, and insanity, it also depends on the circumstances that trigger it. Even if a person experiences negative emotions at a low intensity, exposure to many provoking situations may cause the person to exhibit bullying behavior in cyberspace. Conversely, a person who experiences intense negative psychological feelings might not engage in such behavior if the person encounters only a few stimulating situations [21,26]. Numerous studies have found that negative mental traits—such as frustration, anxiety, and insanity—play a crucial role as causal factors directly and indirectly affecting cyberbullying behavior. This phenomenon arises because individuals often seek to release their inner emotions through various online activities. Some individuals even perceive cyberbullying as a form of amusement when they witness others becoming victims. Furthermore, research reports consistently indicate a concerning trend: cyberbullying is steadily increasing, with young people increasingly considering it a normal occurrence. This serves as a warning sign that all stakeholders must address the issue promptly. Failing to do so could lead to more severe consequences and wider impacts in the long term [7,23,45]. Therefore, negative mental traits including frustration, anxiety, and insanity directly influence youth cyberbullying behavior.

**Figure 1:**
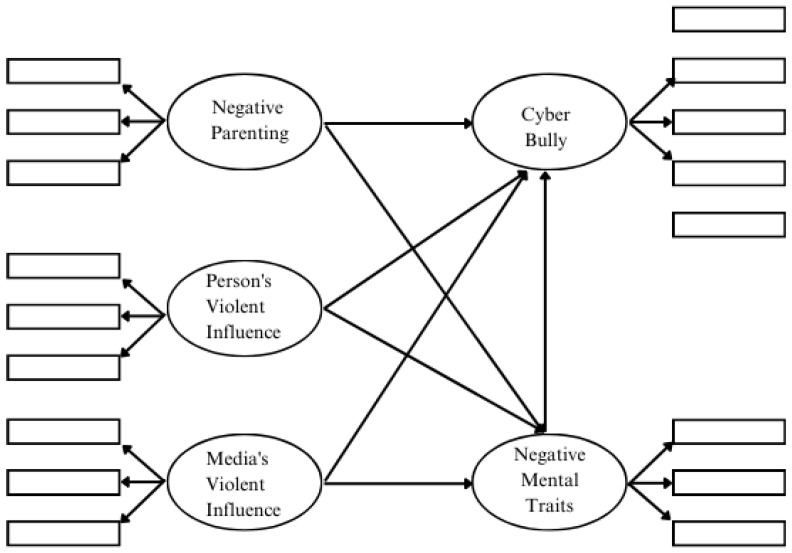
Conceptual framework.

The conceptual framework is shown in Figure 1.

## 3. Materials and Methods

### 3.1. The Subjects

The subjects consisted of youth whose online behavior involved using social media for at least three consecutive hours per day due to the prevailing trend of online cyberbullying [46]. As a general guideline, 340 subjects were recruited, following the recommendation by multilevel analysts, which suggests 20 subjects per variable [47]. The sampling process followed a multistep approach: Step 1: District Selection. Districts were chosen through stratified random sampling based on three levels: Red districts: high densities of losses per capita; pink districts: medium per capita loss density; and green districts: low densities of losses per population. The selection criteria were based on data and trends in violence from the Center of Deep South Watch [48]. Then 1 district/province was selected per one region through simple random sampling and 9 districts were ultimately selected. Step 2: Sub-District Selection. Within each district, two sub-districts were chosen using a simple random sample selection method (lots drawn without replacement). This resulted in a total of 18 sub-districts. Step 3: Final step of sample section. In the final step, 18 or 19 subjects per sub-district were selected, yielding a total of 340 individuals. The subject was identified in collaboration with the sub-district youth group of the local government organization. Their assistance was sought to coordinate and jointly select youth participants for this research.

### 3.2. Research Instruments

The research instrument comprised a 6-part questionnaire developed from scratch by the researcher (original in Thai). This questionnaire was created through an in-depth study of related concepts, theories, and relevant literature. Its purpose was to define operational terms and establish the structure of the variables under investigation. The question items were formulated based on the operational definitions, and relevant questions from existing academic sources were adapted. Then, the questionnaire was validated by 5 experts for content validity resulting in IOC (item–objective congruence) values ranging from 0.60 to 1.00. A pilot test was conducted with 45 nonsample participants and the internal consistency was measured using Cronbach’s alpha coefficient, yielding a value of 0.74–0.85.

### 3.3. Measures

Cyberbullying is a self-report questionnaire that measures the tendency to cyberbully others in the past year. It consists of 24 items scored on a five-point rating scale: 5 = Very Often, 4 = Regularly, 3 = Sometimes, 2 = Once or Twice, and 1 = Never. This questionnaire divided cyberbullying into five aspects as follows: (1) Gossiping and revile in the cyber world with 5 items, for example, “I harmfully gossip in the cyber world”. (2) Cyber defamation with 5 items, such as “I post defamation on cyberspace”. (3) Impersonating others’ names in the cyber world with 5 items, for instance, “I secretly used someone else’s name to fake an image on cyberspace”. (4) Revealing secrets in the cyber world with 5 items, such as “I reveal others’ inferiority complexes in the cyber world”. (5) Deleting or blocking others on cyberspace with 4 items, for example, “I cyber-block others I don’t like from groups”. The internal consistency of the cyberbullying questionnaire was 0.83.

Negative mental traits is a five-point rating scale of the self-report questionnaire with 15 items. The criteria for scoring from “Very Often”, “Regularly”, “Sometimes”, “Once or Twice”, and “Never” were 5, 4, 3, 2, and 1, respectively. This questionnaire contains three subscales: (1) Frustration with 5 items, for example, “I feel uncomfortable when parents monitor my social media use”. (2) Anxiety with 5 items, such as “I am nervous about sharing my friends’ information through social networks”. (3) Insanity with 5 items, for instance, “I sent a sexually explicit image to a friend through a social network”. The alpha coefficient was found to be 0.85.

Negative upbringing is a self-report questionnaire comprising 15 questions that score on a five-point rating scale: “Very Often = 5”, “Regularly = 4”, “Sometimes = 3”, “Once or Twice = 1”, and “Never = 1”. The negative upbringing questionnaires contain three subscales: (1) Controlling upbringing with 5 items, for example, “my parents force the use of social networks only at home and at specified times”. (2) Neglectful upbringing with 5 items, such as “my parents often left me alone to use social networks”. (3) Indulgent upbringing with 5 items, for instance, “my parents support the costs of using all types of social networks”. The Cronbach’s alpha coefficient was 0.78.

Influence personal violence is a 15-question self-report questionnaire with a 5-point rating scale: 1 = Never, 2 = Once or Twice, 3 = Sometimes, 4 = Regularly, and 5 = Very Often. This questionnaire consists of three aspects: (1) Parental violence with 5 items, for example, “my parents violently quarrel with each other through social networks”. (2) Peer violence with 5 items, for instance, “my friends often argue violently over social networks”. (3) Net idols’ violence with 5 items, such as “net idols expose the use of violence through online social networks”. The reliability test of this questionnaire found that the Cronbach alpha was 0.76.

Influence media violence is a self-report questionnaire comprising 15 items that score on a 5-point rating scale. Scores of 1, 2, 3, 4, and 5 were considered as Never, Once or Twice, Sometimes, Regularly, and Very Often, respectively. The influence media violence questionnaires contain three aspects: (1) Film violence with 5 items, such as “I like to watch movies about war and murder on social media”. (2) Gaming violence with 5 items, for instance, “I like to play online games that involve fighting and punching”. (3) News violence with 5 items, for example, “I watched the news of the murder on social media”. The Cronbach’s alpha of the questionnaire was 0.74.

### 3.4. Data Collection

This study used a cross-sectional study design involving surveys of the youth groups of the local government organization in a sample selected from the target population studied, during February–March 2024. The research was reviewed and approved by the Institutional Review Board for Human Subjects Research at Sirindhorn College of Public Health, Nakhon Si Thammarat, under certificate No. E09/2566, on 25 December 2023. Verbal consent to participate in the study was obtained from youth after assurance of confidentiality was given to individuals and group administered. The data collection process was conducted by the researcher and research assistants with experience from many studies who went into the field (the local government organization) together to collect relevant data. A team of data collection staff received training on the field data collection methods and gained a comprehensive understanding of the questionnaire’s specific questions. This training aimed to ensure consistency among the staff members during data collection.

### 3.5. Data Analysis

The analysis of causal factors impacting cyberbullying among youth in the Thailand’s deep south utilized structural equation modeling (SEM) statistical techniques. The objective was to assess the alignment between the hypothesized model and empirical data. Additionally, both direct and indirect influences, and the total impact of causal variables were analyzed. For parameter estimation, the maximum likelihood estimates (ML) method was used to analyze the model based on the specified hypothesis.

## 4. Results

The subjects in this study were 340 youths in the deep south of Thailand, consisting of 220 males and 120 females. The subjects were divided by age range: 22–23 years old (40.6%), 24–25 years old (26.8%), 18–19 years old (17.1%), and 18–19 years old (15.5%).

When considering the skewness or asymmetry of the overall distribution, it was found that all variables were distributed in a left-skewed manner (SK < 0), indicating that the data for all variables had scores higher than the average, with a skewness value between −0.82 and −0.08. When considering the kurtosis or height of the distribution, it was found that all variables had lower kurtosis values than normal. The calculated kurtosis value was less than 3 (KU < 3), indicating that the data for the observed variables were distributed in a relatively flat manner, in which the data of the observed variables are very dispersed. The kurtosis value was between −1.17 and 0.27. Therefore, when considering the skewness and kurtosis values, it was found that the skewness and kurtosis values were slightly different from zero. But they were considered close to zero. Therefore, it was assumed that the observed variables had a normal distribution (see Table 1).

The examination of the consistency of the structural equation model regarding causal factors affecting youth cyberbullying in Thailand’s deep south revealed that the model did not align well with the empirical data. Consequently, adjustments were made by modifying the correlation pairs associated with insignificant error values. Subsequently, the final structural equation model for causal factors demonstrated good alignment with the empirical data. Key statistical indicators include a relative chi-square (χ^2^/df) value of 1.77, a GFI (goodness of fit index) value of 0.94, and a root-mean-square index of approximate differences (RMSEA) of 0.049 (see Table 2).

When examining the results of estimating the direct influence of negative mental traits, it was observed that the negative upbringing variable collectively influenced these traits. The influence of personal violence and the influence of media violence were statistically significant at the 0.001 and 0.05 levels, respectively. Among these variables, the influence of media violence exhibited the highest direct influence on negative mental traits, with a total influence size of 0.81. Following closely was the variable related to negative upbringing, which had a total influence size of 0.49. Lastly, the influence of personal violence had a total influence size of 0.19.

When considering the results of estimating the direct and indirect influences on cyberbullying, it was found that cyberbullying received total influence from variables related to negative mental traits, negative upbringing, the influence of personal violence, and the influence of media violence—all statistically significant at the 0.001 level. The variables with the highest direct and indirect influence on cyberbullying were negative upbringing, with a total influence size of 1.13; the influence of personal violence, with a total influence size of 0.74; and the influence of media violence, with a total influence size of 0.64. Additionally, the variable of negative mental traits had a direct influence on cyberbullying at a statistical significance level of 0.05, with a total influence size of 0.17.

The analysis results regarding the direct and indirect influences, as well as the total influence of latent variables affecting cyberbullying among youth in the three Southern border provinces, are as follows:

(1) Negative upbringing had a total influence on negative mental traits equal to 0.49, which was a direct influence, and negative upbringing had a total influence on cyberbullying equal to 1.13, which was a direct influence equal to 1.21, and as an indirect influence through negative mental traits equal to—0.08.

(2) The total influence of personal violence on negative mental traits was 0.19, which was a direct influence, and the influence of personal violence had a total influence on cyberbullying equal to 0.74, which was a direct influence equal to 0.77 and an indirect influence through negative mental traits equal to—0.03.

(3) The influence of media violence had a total negative influence of 0.81, which was a direct influence, and the influence of media violence had a total influence on cyberbullying equal to 0.64, which was a direct influence equal to 0.78 and as an indirect influence through negative mental traits equal to—0.14.

(4) Negative mental traits had a total influence on cyberbullying equal to 0.17, which was a direct influence.

**Figure 2:**
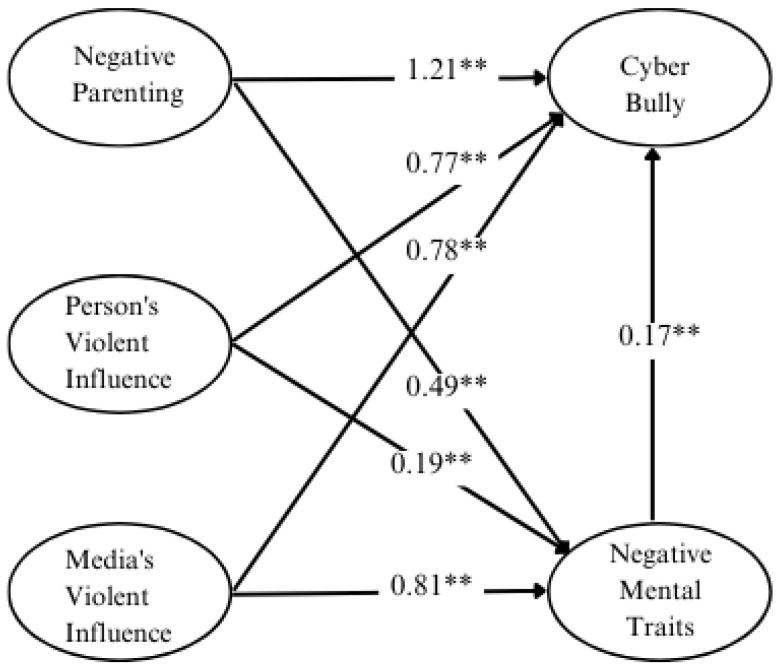
Causal factors affecting youth cyberbullying in the three Southern border provinces. ** *p* < 0.01.

When assessing the ability of latent variables to explain the variation in indicators (or the prediction coefficient, R^2^) within the internal latent variable structural equation, it was found that the prediction coefficient (R^2^) of negative mental traits was equal to 0.892. This indicated that the variables in the structural equation model were able to explain 89.2% of the variance in negative mental traits, while the predictive coefficient (R^2^) of cyberbullying was 0.923. This indicates that the variables in the structural equation model could explain 92.3% of the variance in cyberbullying. Details are shown in Table 3 and Figure 2.

## 5. Discussion

This study found that the most significant direct and indirect influences related to cyberbullying stem from negative upbringing variables. Specifically, family factors such as controlling parenting, neglectful parenting, and indulgent upbringing play crucial roles as causal factors that influence youth to engage in cyberbullying behavior. This behavior stems from a negative upbringing, where parents are controlling, authoritarian, and have neglectful tendencies. By imposing their own opinions and disregarding their child’s opinions, parents may make their children feel dependent, unable to express themselves freely, and constrained by rigid discipline. Violations of these rules result in severe punishment. Consequently, some children alleviate these emotions by engaging in cyberbullying as a means of self-expression [19,20]. In addition, a negative upbringing can lead children to become repressed, mistrustful of others, view others as adversaries rather than friends, adopt a pessimistic outlook, struggle to adapt to society, and exhibit aggression, stubbornness, conflict, uncooperativeness, jealousy, indifference, unruliness, and an inability to get along with others [23,25,27]. When children experience inappropriate parenting, they may express undesirable behavior based on what they have learned. This is particularly true if they perceive that their parents treat them harshly and unfairly, withholding genuine love and support. In such cases, children often react strongly to their parents. Additionally, if they perceive that someone condones their aggressive bullying behavior, they are more likely to repeat such severe actions [26,28]. Therefore, upbringing significantly influences children’s personality, temperament, behavior, and overall development. When children receive an unsuitable upbringing that does not align with their circumstances, it can lead to the development of an undesirable personality that contradicts social norms. Ultimately, this can pose challenges for children in terms of social adjustment and may result in the expression of unwanted behaviors [49,50]. These results are partially supportive of some studies, which found that family upbringing styles have a significant relationship to cyberbullying behaviors among youth [51]. The main factor influencing youth’s cyberbullying behaviors was parental control. Authoritative parenting is a risk factor that is increasingly likely to cause youth to feel irritable, angry, depressed, and frustrated. It also increases youth’s feelings of depression and mental complexity. Ultimately, it causes youth to display undesirable behaviors, especially cyberbullying others. This factor directly and indirectly affects cyberbullying behaviors and negative mental traits [36,52].

The latent variables that directly and indirectly influence cyberbullying include personal violence and the impact of media. This demonstrates that various forms of violence such as parental violence, peer violence, net idols’ influence, film violence, gaming violence, and news violence play crucial roles as causal factors in youth cyberbullying. This occurs because the factors influencing individual violence ultimately contribute to negative behavior in youth. Young individuals learn by observing models and absorbing their behavior, which often unknowingly reinforces their violent conduct. Consequently, they may perceive such behavior as normal. Ultimately, young individuals exhibit these behaviors in their daily lives, believing that doing so can address various problems. The models they choose to imitate initially include people they respect, those with whom they have close relationships, favorite role models, and individuals whose interests align with their own [40,41]. Additionally, the influence of personal violence and media violence contributes to negative life experiences for youth. This serves as the starting point for young individuals to exhibit harmful behavior. They are stimulated to display violent conduct based on examples they have witnessed or encountered. As they repeatedly observe such behavior, their motivation to imitate it grows. Unfortunately, this environment places significant stress on young people over a long period. Consequently, they often develop symptoms of rebellion against adults and believe that using force and violence is necessary to gain acceptance and respect [49,53]. The influence of personal violence and media violence also transmits perceptions, principles, practices, attitudes, and values that are inappropriate for youth. This process renders young individuals ill-equipped to peacefully adapt to societal life, resulting in a high rate of conflict, quarrels, and physical harm among peers. Eventually, this escalates into a serious behavioral problem [39]. Youth who have encountered violence from their surroundings and the media are significantly more likely to exhibit cyberbullying behavior compared to those who have not experienced such violence by 4.50 times [25], 7.60 times [53], and 7.11 times [54]. One causal factor contributing to violent acts and delinquency among youth is exposure to violent or illegal events through various media and interactions with people [55]. The results of this research are also consistent with the study that noted that the influence of personal violence and media violence has become a negative role model for youth. Youth often think that the violence they have witnessed can be used to cyberbully others in their daily lives through a process of absorption into their minds [20,24]. Youth have more opportunity to learn by observing the cyberbullying behavior of their closest and most respected people. Those behaviors and people become negative role models, acceptable, and can be followed without any problems [38]. A risk factor that influences youth’s cyberbullying behavior is encounters with both violent incidents from personal violence and media violence. This is because the incident unknowingly promotes youth’s cyberbullying. Thus, personal violence and media violence are closely correlated with cyberbullying behaviors [20,21,40].

Furthermore, negative mental traits also exert both direct and indirect influences on cyberbullying. These negative mental traits such as frustration, anxiety, and feelings of insanity serve as crucial causal factors that influence youth to engage in cyberbullying behavior. The connection lies in the fact that young individuals with negative mental traits often experience emotions stemming from unmet desires. They may feel unable to pursue their heartfelt wishes or achieve their set goals. Youth frequently encounter obstacles, face shortages, and experience failures, leading to frustration, anxiety, and feelings of insanity. As these negative mental traits accumulate, youth seek outlets for these emotions. Ultimately, these emotions become cyberbullying behavior in response to frustration, anxiety, and feelings of insanity [20,27]. Furthermore, negative mental characteristics also influence the emotions, feelings, and minds of youth. As a result, young individuals may experience anger, fear, and repression, along with emotional deficiencies that make them easily irritable and prone to anger. They may express themselves through inappropriate words and actions, feeling that they have not received justice and are not clearly understood. Over time, this accumulation of negative emotions leads to frustration, anxiety, and even feelings of insanity, which in turn can manifest as cyberbullying behavior toward others [26,28]. Hence, it becomes evident that cyberbullying behavior arises from negative mental influence, similar to the pressure building up in a boiling kettle. When the forces of frustration, anxiety, and insanity accumulate, they seek release through inappropriate expressions of behavior [26,28,29]. The root cause of youth’s cyberbullying behavior lies in negative mental traits. However, the extent to which cyberbullying behavior manifests depends on the stimulating situations that trigger it. In other words, there must be a relationship between a person’s internal characteristics and external stimuli. If an individual has few negative mental traits (such as frustration, anxiety, and insanity) but encounters numerous stimulating situations, cyberbullying behavior may occur. Conversely, if someone possesses many negative mental traits but experiences few stimulating situations, cyberbullying behavior may not manifest [26,28,49]. One noticeable characteristic among youth who cyberbully others is their inability to contain emotions such as anger, frustration, anxiety, and even feelings of insanity, which often manifest as undesired behavior [51,56]. Additionally, an individual’s display of cyberbullying behavior frequently depends on the context of whether it is frustration, anger, irritation, stress, or strictness [57,58]. In addition, negative mental traits refer to the emergence of youth’s emotions and feelings whose needs are not being met. It also means being prevented from performing the desired behavior. This is due to encountering various obstacles, which later turn into frustration and suppression. Then, youths choose to vent their feelings on social media by directly cyberbullying them [41,51]. Youth who use cyberbullying often think that the behavior is normal, accepted by society, and can be imitated. These youth often face many emotional problems, especially concerning repression, leading to emotional confusion, frustration, depression, and self-destructive tendencies. Negative mental traits are a psychological state that leads to direct cyberbullying [7,20,59].

## 6. Conclusions

Cyberbullying received total influence from variables related to negative mental traits, negative upbringing, the influence of personal violence, and the influence of media violence—all statistically significant at the 0.001 level. The variables with direct and indirect influence on cyberbullying were negative upbringing, the influence of personal violence, and the influence of media violence, with a total influence size of 1.13, 1.13, and 0.64 respectively. Additionally, the variable of negative mental traits had a direct influence on cyberbullying at a statistical significance level of 0.05, with a total influence size of 0.17.

The findings from this research hold value for relevant organizations, institutions, and individuals, helping in the formulation of effective policies and concrete strategies to prevent, monitor, address, and reduce cyberbullying among youth. To achieve this, awareness and priority should be given to negative upbringing factors, which exert the most significant influence, followed by personal and media influences. These considerations serve as essential guidelines for preventing youth cyberbullying before it escalates into a more serious societal problem. This research is the preliminary study on this topic. The result of this study might become the basis information for policymakers to take the cyberbullying issue into full consideration and further steps to determine concrete practical strategies for monitoring, preventing problems, solving problems, and reducing problems with true effectiveness and efficiency. However, this research still has some limitations. Firstly, the sample selection might be too narrow, as the data collection area was limited to the youth groups of the local government organization. Secondly, this research only focused on youth whose online behavior involved using social media for at least three consecutive hours per day due to the prevailing trend of online cyberbullying. Further research about cyberbullying in Thailand’s deep south and Thailand is encouraged to examine the situation deeply and provide more information to help overcome cyberbullying behavior issues. In addition, there should be serious research and development of programs to prevent and reduce cyberbullying behavior and create an integrated network of cooperation to solve the concrete problem of cyberbullying.

## Figures and Tables

**Table 1:** Normal distributions of variables.

Variables	$\bar{Χ}$	S.D.	KU	SK
Cyberbullying	4.59	0.18	−0.33	−0.60
Gossiping and revile in the cyber world	4.68	0.22	0.27	−0.69
Cyber defamation	4.42	0.29	−0.22	−0.30
Impersonating others’ names in the cyber world	4.60	0.24	−0.61	−0.22
Revealing secrets in the cyber world	4.68	0.24	0.24	−0.82
Deleting or blocking others on cyberspace	4.54	0.26	−0.40	−0.18
Negative mental traits	4.48	0.26	−1.17	−0.12
Frustration	4.58	0.27	−0.61	−0.49
Anxiety	4.46	0.28	−0.51	−0.10
insanity	4.41	0.34	−0.79	−0.36
Negative upbringing	4.51	0.22	−0.26	−0.30
Controlling upbringing	4.43	0.27	−0.59	−0.34
Neglectful upbringing	4.53	0.30	−0.51	−0.20
Indulgent upbringing	4.58	0.28	−0.59	−0.36
Influence personal violence	4.55	0.24	−0.60	−0.58
Parental violence	4.70	0.23	−0.96	−0.28
Peer violence	4.47	0.34	−0.16	−0.53
Net idols’ violence	4.49	0.28	−0.74	−0.17
Influence media violence	4.53	0.20	−1.10	−0.13
Film violence	4.57	0.25	−0.50	−0.41
Gaming violence	4.43	0.29	−0.75	−0.08
News violence	4.57	0.20	−0.29	−0.31

**Table 2:** Consistent index of hypothesis model and empirical data.

Index Value	Criteria	Before Adjusting Model		After Adjusting Model	
		Statistics	Result	Statistics	Result
χ^2^/df	≤2.00	223.02/109 = 2.05	Not consistent	187.54/106 = 1.77	Consistent
CFI	≥0.90	0.94	Consistent	0.95	Consistent
GFI	≥0.90	0.93	Consistent	0.94	Consistent
AGFI	≥0.90	0.90	Consistent	0.91	Consistent
RMSEA	≤0.05	0.052	Not consistent	0.049	Consistent
SRMR	≤0.05	0.066	Not consistent	0.048	Consistent

**Table 3:** Causal factors affecting youth cyberbullying in the three Southern border provinces.

	Negative Mental Traits			Cyberbullying		
	DE	IE	TE	DE	IE	TE
Negative upbringing	0.49 ***	--	0.49 ***	1.21 ***	−0.08 *	1.13 ***
The influence of personal violence	0.19 *	--	0.19 *	0.77 ***	−0.03 *	0.74 ***
The influence of media violence	0.81 ***	--	0.81 ***	0.78 ***	−0.14 *	0.64 ***
Negative mental traits	--	--	--	0.17*	--	0.17 *
	R-Square (R^2^) = 0.892			R-Square (R^2^) = 0.923		

*** *p* < 0.001, * *p* < 0.05.

## Data Availability

Data availability is restricted due to privacy reasons. However, data may be available by writing to the corresponding author.
